# Embedding a Sensitive Liquid-Core Waveguide UV Detector into an HPLC-UV System for Simultaneous Quantification of Differently Dosed Active Ingredients during Drug Release

**DOI:** 10.3390/pharmaceutics14030639

**Published:** 2022-03-14

**Authors:** Rebecca Chamberlain, Hellen Windolf, Bjoern B. Burckhardt, Jörg Breitkreutz, Björn Fischer

**Affiliations:** 1 Institute of Pharmaceutics and Biopharmaceutics, Heinrich Heine University, Universitätsstraße 1, 40225 Düsseldorf, Germany; rebecca.chamberlain@hhu.de (R.C.); hellen.windolf@hhu.de (H.W.); joerg.breitkreutz@hhu.de (J.B.); 2 Institute of Clinical Pharmacy and Pharmacotherapy, Heinrich Heine University, Universitätsstraße 1, 40225 Düsseldorf, Germany; bjoern.burckhardt@hhu.de

**Keywords:** liquid-core waveguide, hot melt extrusion, low-dosed dosage forms, analytics of extruded filaments, fused filament 3D printing, oral dosage form, personalized medicine

## Abstract

Individual dosing of pharmaceutics and personalized medicine have become important with regard to therapeutic safety. Dose adjustments, biorelevant drug release and combination of multiple active substances in one dosage form for the reduction in polymedication are essential aspects that increase the safety and acceptance of the patient’s pharmacotherapy. Therefore, not only innovative drug products but also new analytical methods are needed during the drug development phase and for quality control that can simultaneously determine different active ingredients and cover wide concentration ranges. We investigated a liquid-core waveguide UV absorbance flow cell detector coupled to an existing HPLC-UV system. A Teflon AF 2400 capillary tubing of 20 cm length was connected in series to the HPLC flow line and enabled a lower limit of quantification of 1 ng/mL pramipexole (increase in sensitivity by 20 compared to common 0.9 cm flow cells). This allowed the low-concentration of pramipexole and the higher concentrations of levodopa and benserazide occurring during drug release to be determined in a single chromatographic run within 22.5 min.

## 1. Introduction

Current therapy guidelines for the treatment of non-communicable diseases (e.g., ESC guideline on hypertension) have recently changed their recommendations based on the latest scientific data towards a dual combination at the initiation of therapy [1]. A rational extension of this approach is to offer such dual or multiple combinations directly in one dosage form to increase patient adherence by reducing the number of drug products to be taken [2]. According to the FDA, a combination product is defined as a dosage form containing two or more drugs in a single pill [3]. Not only is the combination of drugs of interest, but personalized medicine also focuses on individual dosing for each patient in terms of age, weight and comorbidities. The “one-size-fits-all” approach of APIs that have a narrow therapeutic window and therefore risk side effects with small differences in dosage is in the process of being replaced by new approaches, e.g., 3D printing of medicines [4]. With 3D printing it is possible to fabricate complex geometries that incorporate multiple APIs with diverse release kinetics [5,6,7,8]. This potentially allows various active ingredients to be combined in a single 3D printed tablet. In previous studies, we found that even if the dose is varied, the release profile remains the same if the surface area to volume ratio is kept constant [9]. In analyzing release kinetics of all incorporated APIs especially during the development phase, analytical methods that can quantify all incorporated APIs simultaneously are useful. To ensure the detection of a low concentration of a high-potent drug during drug release from its dosage form, a sensitive analytical method must be selected, the dissolution apparatus can be adjusted, or the volume of the medium in the dissolution vessel can be reduced [10,11,12,13,14]. Wang et al. found that dissolution studies conducted in mini vessel led to the same results as in a vessel prescribed by European Pharmacopeia, but the paddle speed had to be increased [15]. Mini paddle apparatus is recommended by Klein et al. to be used for powders, multiparticulate dosage forms and small tablets or capsules [16]. However, it is important that the dissolution study proceeds under sink conditions to avoid influencing the drug release behavior of the corresponding API, which would no longer be the case with high-concentrated APIs in a combination product [17]. The combination of high-performance liquid chromatography UV detection with mass spectrometry (MS) detection and high dynamic range diode array detection (high dynamic range DAD) is capable of covering a wide concentration range [18,19]. For a cost-effective and easy-to-integrate detection system, liquid-core waveguide detection systems are used and described in literature by several research groups. Li et al. have developed a portable setup with extended light path that can be used to detect very low concentrations [20]. A modified detection system compared to the detection system used in this work with a charged-coupled device including optical fibers and a liquid-core waveguide was utilized by Kottke et al. to detect low concentrations of desmopressin during permeation studies. However, they used fluorescence measurements to be able to detect 9.5 ng/mL of desmopressin, a method that was tenfold more sensitive in comparison with reference HPLC detection systems [21]. In this study, drug preparations of three different APIs were analyzed with substantially different dosage ranges. Levodopa (LD), the precursor to the neurotransmitter dopamine, is used in the clinical treatment of Parkinson’s disease in a single dose with oral administration of 25–200 mg [22]. The decarboxylase inhibitor benserazide (BZ) is routinely administered orally in combination with levodopa in a ratio of 1:4 (BZ/LD) to prevent peripheral degradation of levodopa [23]. This results in a dosage range between 12.5 and 50 mg for benserazide. The dopamine agonist, pramipexole, is administered at a lower drug amount. Here, only 0.088–3.15° mg is required for pharmacologically efficient drug concentrations after oral administration [24]. The combination of these three APIs should reduce the number of tablets for Parkinson’s patients and guarantee individualized dosing. The fluorescence detection cannot be applied as pramipexole does not show fluorescence in aqueous medium, which leads to an exclusion of the detector selection option. Consequently, UV measurement of pramipexole was favored, and the suitability of detection by a liquid-core waveguide ultraviolet absorbance detection system (LCW-UV) was investigated, which is frequently used in literature [25,26,27,28,29,30,31,32]. The extended detection path of the LCW should result in the detection range of UV measurement being extended into the low concentration range (minimum of 1 ng/mL pramipexole) while at the same time also allowing APIs to be detected in the higher concentration range (maximum of 2 µg/mL levodopa) by the UV detector of the HPLC covering a difference in concentration by a factor of 2000. 

## 2. Materials and Methods

### 2.1. Experimental Procedure of Hot Melt Extrusion Runs

Poly-(ethylene-vinyl acetate)-copolymer (82:18) (EVA; Escorene^®^ FL01418, TER Chemicals, Hamburg, Germany) and polyvinyl alcohol (PVA; Parteck MXP^®^, Merck, Darmstadt, Germany) were used as polymer matrices. Pramipexole (P, Chr. Olesen, Denmark), levodopa (Zhejiang Wild Wind Pharmaceutical, Dongyang, China) and benserazide (s.p. quimica, s.a., Barcelona, Spain) were used as model drugs. PVA was chosen as a matrix for pramipexole, and EVA was used for the matrix of the fixed combination of levodopa and benserazide within one filament. Poly-(vinylpyrrolidone-vinyl acetate)-copolymer (60:40) (VA; Kollidon VA 64^®^, BASF, Ludwigshafen, Germany) was incorporated into formulation 2 to act as a pore-forming agent. The composition of the two formulations is listed in Table 1.

All filaments were prepared by hot melt extrusion (HME) with a co-rotating twin-screw extruder with a hot-melt extrusion die (Pharmalab HME 16, Thermo Fisher Scientific, Rockford, IL, USA). A gravimetric feeder (K-SFS-24/6, Coperion K-Tron, Niederlenz, Switzerland) was used for all experiments. A vent port was set between kneading zones 1 and 2 for all extrusions. An in-house manufactured die with a diameter of 1.85 mm was used. The desired filament diameter was achieved using a belt hauled-off unit of a winder (Brabender, Duisburg, Germany) with a belt speed of 0.8 m/min, and the filament was pulled through a roll-system with four 360° air flow ring nozzles (Super Air Wipe, Exair, Cincinnati, OH, USA) for active cooling of the melt. With a laser-based diameter measurement module (Laser 2025 T, Sikora, Bremen, Germany), we continuously measured and logged the filament diameter during the process with a readout rate of 1 Hz to ensure diameter homogeneity [33] of PVA-P and EVA-LD-BZ filaments. The screw speed was set to 20 rpm and powder feed rate was set to 2 g/min. The screw configuration and the temperatures of the heating zones were set according to the physical properties of the polymers for both formulations and are summarized in Table 2. 

### 2.2. Dissolution Testing

**Figure 1:**
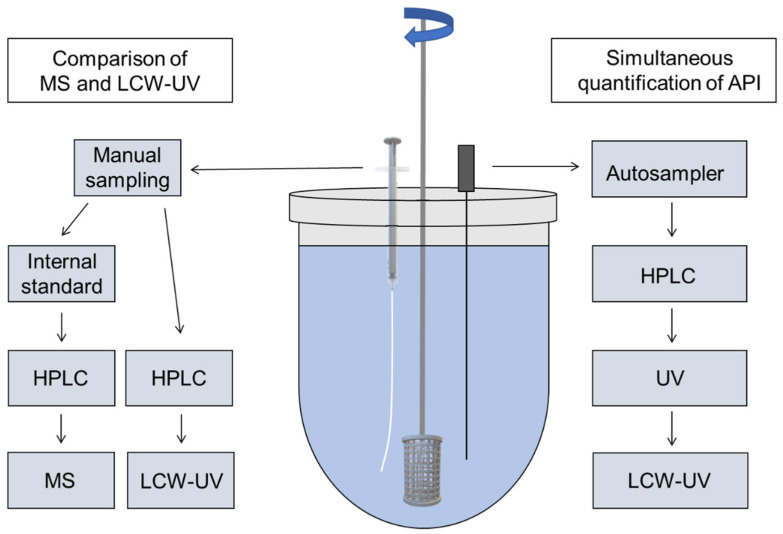
Modified dissolution testing with 3D printed basket based on Ph. Eur. monographs 2.9.3 and 5.17.1 with manual sampling (**left side**) for MS and LCW measurement of single pramipexole filaments and automatic sampling (**right side**) for the simultaneous content determination of three APIs (pramipexole, benserazide and levodopa) during dissolution by HPLC-UV and LCW-UV.

According to European Pharmacopoeia monographs 2.9.3 and 5.17.1, release studies were performed with the basket method (method 1) in a dissolution tester (DT 700, Erweka, Langen, Germany) [13,34]. The baskets were 3D printed from water insoluble polylactide acid. They had to be adapted for extruded filaments, since the mesh size of the regular Ph. Eur. baskets is small, and the baskets were clogged by the swollen polymer of the PVA formulation [35]. This affected the hydrodynamic medium flow around the filament. Vessels contained 1000 mL of degassed 0.1 N hydrochloric acid at pH 1.2 at a temperature of 37 ± 0.5 °C, and the baskets were stirred at 50 rpm. Dissolution tests were performed under sink conditions of pramipexole (c_s_ = 200 mg/mL [36]; maximum concentration 0.1 µg/mL). For the comparison of released pramipexole from PVA matrix by MS vs. LCW-UV detection, 5 mL samples were taken from the dissolution medium after 5, 10, 15, 30, 45, 60, 90 and 120 min by a syringe. One 2.5 mL aliquot was used to fill HPLC vials for UV measurement, and another 2.5 mL was poured into a 96-well plate for mass spectrometric analysis and mixed with 50 µL of internal standard (Figure 1, left side). No replenishment was conducted, but the amount of removed liquid volume by sampling was calculated for the corresponding subsequent sampling time point. The same dissolution testing setup was used for the simultaneous analysis of the PVA-P filament and EVA-LD-BZ filament, but samples were obtained by an autosampler (Vision^®^ AutoFill™ + AutoPlus™, Teledyne Hanson Research, Chatsworth, CA, USA). The content of P, LD and BZ was analyzed by UV and LCW-UV detection (Figure 1, right side). To avoid oxidation processes of BZ, the whole dissolution tester was wrapped in aluminum foil for light protection, whenever filaments were released containing BZ [37]. 

The dissolution tests were also performed under sink conditions of levodopa (c_s_ = 12 mg/mL [38]; maximum concentration 0.1 mg/mL) and benserazide (c_s_ = 33.5 mg/mL; maximum concentration 0.025 mg/mL). Samples (1.5 mL) were taken by the autosampler and were filled in HPLC vials every 5 min for the first 30 min, then every 10 min for the next 30 min, followed by sampling at time point 90 and 120 min. After a release time of 120 min, samples were taken every 120 min until 10 h had passed. At every time point, 1.5 mL of 0.1 N hydrochloric acid was returned to the dissolution medium, and the amount of API removed by sampling was calculated for the corresponding subsequent sampling time point.

### 2.3. LC-MS/MS Quantification of Pramipexole during Dissolution 

The mass spectrometric quantification of pramipexole was carried out on an Agilent 1200 Serie HPLC system (Agilent, Ratingen, Germany) coupled to a triple-quadrupole tandem mass spectrometer API 4000 (SCIEX, Vaughan, ON, Canada) with an electrospray ionization (ESI) interface. Samples were injected by a CTC HTC PAL autosampler (CTC Analytics AG, Zwingen, Switzerland) equipped with a 20 μL sample loop. Chromatographic separation was achieved on a Phenomenex Synergi Hydro RP (150.0 × 2.0 mm; 4 µm) column using isocratic condition with acetonitrile and 10 mM ammonium acetate in water as mobile phase (70/30 (*v/v*)). The compound specific parameters were as follows: declustering potential of 66 V/81 V, entrance potential of 8 V, cell entrance potential of 21 V/19 V and cell exit potential of 30 V/6 V. CAD gas was set to 7 psi, while gas 1 and gas 2 were adjusted to 32 and 45 psi, respectively. Curtain gas was 37 psi. Ion spray voltage was set to 5.5 kV with 600 °C ion source temperature. Time between injections was 2 min with retention times of 0.95 min for pramipexole and 1.1 min for talipexole. The mass-to-charge ratio of 212.2 to 153.1 *m*/*z* for pramipexole and 304.1 to 260.0 *m*/*z* for the internal standard talipexole was monitored utilizing multiple reaction monitoring and positive ionization mode. The method was characterized by a linear range from 0.19 to 100 ng/mL (1/x^2^ weighing). The intra-run accuracy varied from −4.5 to 13.2% (n = 3 per quality control level). The collected data were analyzed using Analyst 1.6.2 (Applied Biosystems/MDS SCIEX, Concord, ON, Canada) with IntelliQuan ^®^ as the integration algorithm without smoothing.

### 2.4. Chromatographic Conditions for UV and LCW-UV Measurements

**Figure 2:**
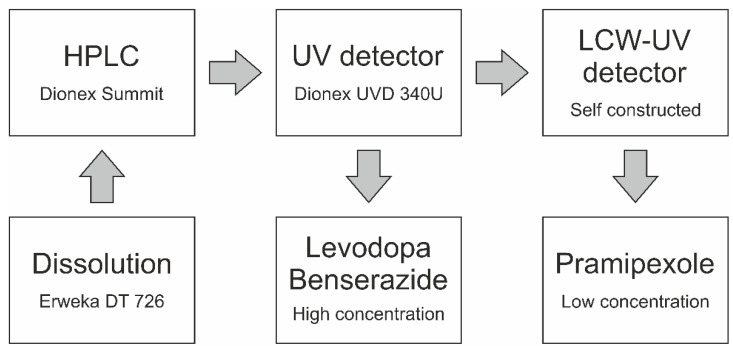
Inline coupling of a UV detector and LCW-UV detector for the simultaneous analysis of pramipexole, levodopa and benserazide.

For dissolution analysis, dissolution medium of pramipexole filaments was analyzed by UV measurements. The HPLC system (Dionex, Sunnyvale, CA, USA) was equipped with a quaternary pump (P 580 A, Dionex, Sunnyvale, CA, USA) and an autosampler (ASI-100, Dionex, Sunnyvale, CA, USA). For the first HPLC method (method 1) analyzing only pramipexole, a 150 × 4.6 mm column (Eurospher II 100-5 C18A, Knauer, Berlin, Germany) with an integrated precolumn was used. The mobile phase consisted of methanol (mobile phase B) and ammonium acetate buffer (0.05 M, pH 4). The flow rate was set to 1 mL/min, the oven temperature was set to 40 °C and the injection volume was 200 µL. The gradient was as follows: mobile phase B was increased from 5 to 95% (*v/v*) within the first 10 min, held for 5 min at 95% (*v/v*) and decreased to 5% (*v/v*) again until 20 min after the sample injection. The column was equilibrated for 3 min before the next sample was injected. Detection was achieved by measuring the UV absorbance of the sample at 264 nm. This UV detector is described as the reference UV detector (UV). Since the release of 88 µg pramipexole from a drug preparation is described (1% of released drug is 0.88 ng/mL), and thus the calibration curve must be extended, a liquid-core waveguide ultraviolet detection system (LCW-UV) was incorporated into the HPLC flow, which is described in Section 2.5. For the second HPLC method (method 2) encompassing all three APIs, a 250 × 4.6 mm column (Eurospher II 100-5 C18A, Knauer, Berlin, Germany) with an integrated precolumn was utilized (Figure 2). The same mobile phase, flow rate and column temperature were used. The gradient was as follows: Mobile phase B was increased from 1 to 5% (*v/v*), within the first min, held at 5% (*v/v*) for 4 min, increased from 5 to 10% (*v/v*) within 1 min, held at 10% (*v/v*) for 4 min, increased again from 10 to 20% (*v/v*) within 1 min, held for 4 min at 20% (*v/v*), increased again from 20 to 99% (*v/v*) within 5 min, held for 2 min at 99% (*v/v*) and decreased to 1% (*v/v*) within 0.5 min and again until 22.5 min after sample injection. An equilibration time of 3.5 min per run was allowed to pass before the next sample was injected. An injection volume of 200 µL was chosen to analyze the APIs during drug dissolution. Again, the second detector system (Section 2.5) was incorporated into the HPLC flow for the quantification of low pramipexole concentrations. Detection was achieved by measuring the absorbance of the sample at 264 nm using the UV detector (BZ, LD) and the LCW-UV detector (P) (Figure 2). 

### 2.5. Liquid-Core Waveguide Ultraviolet Detection System (LCW-UV) 

**Figure 3:**
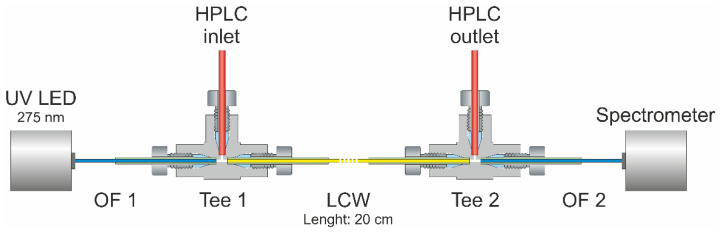
Assembly of the implemented liquid-core waveguide ultraviolet detection system consisting of a UV LED (absorption maximum: 275 nm), two optical fibers (OFs 1 and 2), a 20 cm liquid-core waveguide (LCW), two tee pieces and a charge-coupled device detector.

Figure 3 shows the schematic composition of the LCW-UV detection system. Two stainless steel tees (U-428, 508 µm through hole, IDEX Health & Science, Oak Harbor, WA, USA) were applied to pass the eluent from the HPLC through a 20 cm liquid-core waveguide (LCW, Teflon AF 2400, RI = 1.29, d_inner_ = 200 μm, d_outer_ = 813 μm, Biogeneral, CA, USA). A UV-LED (LED33UV270-6060-100, LG Innotek, Seoul, Korea) was chosen as the light source providing a measured emission maximum at 275 nm. The light was guided into tee 1 at the pigtail end of an optical fiber (d_inner_ = 600 µm, FDP600660710, Laser Components GmbH, Olching, Germany). To avoid breakage of the fiber, the cladding of the fiber was not removed and the front side was optically polished together with the cladding. At the same time, the polymer cladding ensured sufficient tightness of the flow cell. Fluoropolymer sleeves (NanoTight Sleeve Green, d_inner_ = 838 μm, d_outer_ = 1588 μm, IDEX Health & Science, Oak Harbor, WA, USA) were used to match the dimensions between the LCW, the optical fibers and the tees. 

For detection, the light was collected by a second optical fiber connected to tee 2 and guided into a spectrometer (Kymera 328i B2 equipped with Newport diffraction grating 600 L/mm, blaze 300 nm, Oxford Instruments, Abingdon, UK), assembled with a charge-coupled device detector (CCD, iDus DV420A BU2, Oxford Instruments, Abingdon, UK). For spectra acquisition, the exposure time was set to 0.03 s, and the number of accumulations for each spectrum was set to 16, resulting in an acquisition rate of 2.08 Hz. The readout mode was adjusted to full vertical binning, the vertical pixel shift was 16.25 µs and the readout rate was 100 kHz. 

### 2.6. Mathematical Description

The definition of absorbance is:

(1) $$A=\log\left(\frac{I_{0}}{I}\right)=\varepsilon \;\times \;b\;\times \;c$$

*A* is the absorbance, $I_{0}$ is the incident intensity, I transmitted intensity, ε is the molar absorptivity, *b* is the path length and *c* is the molar concentration [39]. By increasing the light path *b* using a liquid-core waveguide, the absorbance *A* of pramipexole was increased, which was particularly important to be able to detect the low concentrations during dissolution testing. 

Thus, in the first step, the LCW-UV setup was compared with an established method (mass spectrometry) for drug release of low-dosed filaments. To evaluate the similarity of the release curves measured by two different analytical methods, the mean dissolution time (MDT) and the similarity factor (*f*_2_-value) were calculated [40,41].

(2) $$\mathrm{MDT}=\frac{ABC}{c_{\infty }}=\frac{\sum_{i=0}^{\infty }\left[\left(c_{i+1}-c_{i}\right)\times \frac{t_{i}+t_{i+1}}{2}\right]}{c_{\infty }}$$

*ABC* stands for the area between the curves and is calculated via the trapezoidal equation with *c* as the concentration of the API released over time *t* and $c_{\infty }$ as the initial drug load of the filament.

(3) $$f_{2}=50\times \log\left\{\left[1+\frac{1}{n}\overset{n}{\underset{t=1}{\sum }}\left(R_{t}-T_{t}\right)²\right]\times 100\right\}$$

In this equation, $R_{t}$ and $T_{t}$ stand for the mean released amounts of the API in % at time point t of the reference (MS result) and the test method (LCW-UV result) and *n* for the number of time points. A $f_{2}$-value around 100 is desired, which indicates that the curves are identical. A value of 50 or more is accepted, which indicates that the values differ by a maximum of 10%. Values below 50 indicate that the curves can no longer be considered similar [34]. 

## 3. Results and Discussion

### 3.1. Spectroscopic Evaluation of LCW-UV Measurements

**Figure 4:**
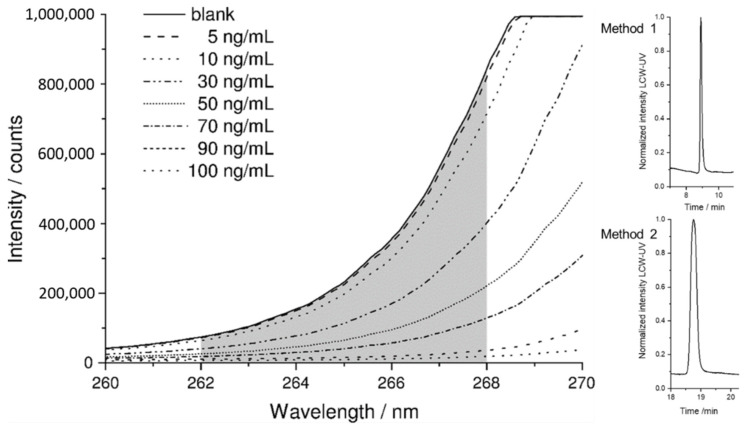
Measured intensity of a blank sample and of the peaks of various concentrations of pramipexole (5–100 ng/mL) with highlighted integration range between 262 and 268 nm (grey). Resulting pramipexole peaks (100 ng/mL) after integration for methods 1 and 2.

A small range of the rising slope of the UV LED was used for the absorbance measurement. The grating of the spectrometer was adjusted so that the CCD detector was in saturation from 268.5 nm upwards. The evaluation was performed between 262 and 268 nm. With these settings, the lowest LLOQ could be achieved. The raw spectrum of the blank (pure mobile phase) is displayed in Figure 4*,* where the detector depicts the highest signal, since the eluent shows hardly any absorbance at the evaluated wavelength range. As the concentration of pramipexole increases, the signal intensity decreases because the API is absorbing light originating from the UV-LED. The corresponding signals in the peak maximum of a concentration series of 5–100 ng are also shown Figure 4. To obtain a chromatogram from raw data of the measured intensity, spectra were integrated between 262 and 268 nm and evaluated with respect to the measurement time. The obtained peaks of pramipexole can be identified in the chromatogram obtained by LCW-UV measurement after 8.9 (method 1) and 19.3 min (method 2). These settings were consequently used for spectroscopic evaluation of LCW-UV detection systems for calibration curves and for the quantification of pramipexole during drug release.

### 3.2. MS and LCW-UV Measurement in Comparison

**Figure 5:**
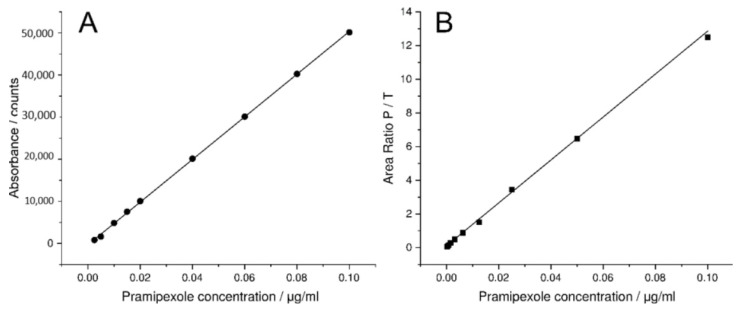
Calibration curve of LCW-UV detection system (**A**) of pramipexole (2.5–100 ng/mL) and calibration curve of MS analysis (**B**), where the ratio of the areas of pramipexole and talipexole (area ratio P/T) were plotted against the concentration of pramipexole (0.19–100 ng/mL).

**Figure 6:**
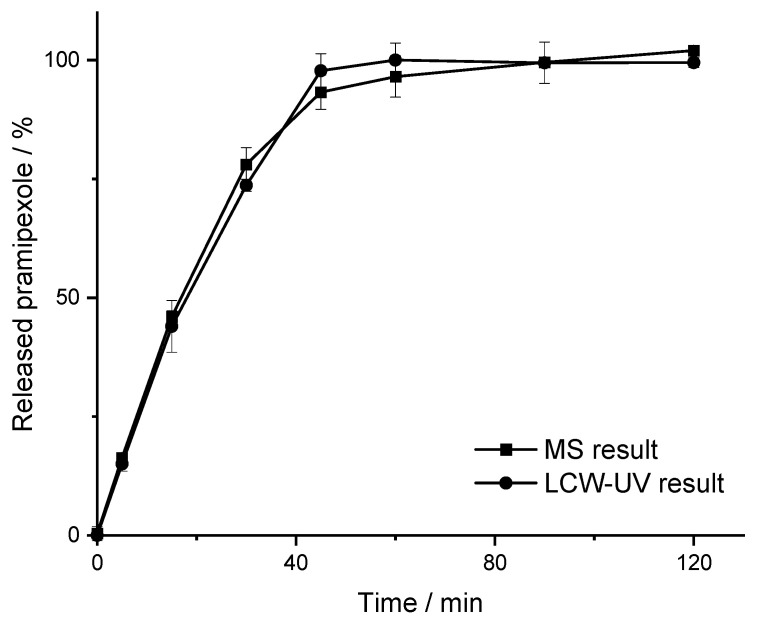
Release curve of 0.5% (*w/w*) pramipexole filaments ($\bar{m}$ = 17.61 mg) determined by MS and LCW-UV measurement (*n* = 3, $\bar{x}\;\pm$ SD).

For mass spectrometric measurements of pramipexole, an internal standard with a known concentration was used, which served to give a ratio of the signal intensity. Thus, the ratio of the areas of pramipexole and talipexole (area ratio P/T) was used for the calibration curve obtained by MS measurements. A linear range from 0.19 to 100 ng/mL (Figure 5B) was found; however, concentrations below 1 ng/mL were not relevant for the release study, since a higher concentration of pramipexole is already reached in the vessel after the first sample draw after 5 min. UV detection was able to quantify a concentration of 0.05 µg/mL (lower limit of quantification (LLOQ): 50 ng/mL with an S/N = 11) pramipexole, which for samples containing 88 µg pramipexole in 1000 mL release volume means that the release curve could only be described after a drug release of more than 56% (method 1). Thus, by using the LCW-UV detection system, the linear range of the UV investigation was extended, and a concentration of 2.5 ng/mL could be quantified (LLOQ: 2.5 ng/mL with S/N = 10). Applying the LCW-UV measurement, the detection limit was improved by a factor of 20, resulting in the ability to describe the drug release curve of low-dosed pramipexole preparations after a drug release of 2.5 ng/mL (2.8% API release). This enhancement of the detection limit only results from the extended light path evoked from the LCW, which is described by the Beer–Lambert law Equation (1). However, the measurements showed that the LCW has an upper limit of quantification at 100 ng/mL. Therefore, concentrations of pramipexole during drug release between 2.5 and 100 ng/mL (Figure 5A) can be determined by LCW-UV measurements, and concentrations above 100 ng/mL would need to be quantified by UV measurements. Since both methods have an overlapping linear range between 50 and 100 ng/mL, the evaluation of these concentrations of pramipexole could be depicted by both detection methods. After the calibration curve ranges of both methods were established, a drug release study was performed as described in Section 2.2. The dissolution profile of 0.5% (*w/w*) pramipexole filaments, which were manufactured by HME (Section 2.1) and were collected in equilibrium condition for drug content [42], resulting from dissolution testing in 1000 mL of 0.1 N HCl, are shown in Figure 6.

The curves indicated that the results for MS and the LCW-UV result in a comparable dissolution profile. For MS results, the MDT is 20.38 min, and the MDT of the dissolution curve obtained by LCW-UV results is 19.73 min (Equation (2)). This results in a minimal discrepancy of the MDT in 33 s, which appears negligible. Since the calculated *f*_2_-value corresponds to 92, it was assumed that the analysis with LCW-UV would show similar results to the investigation with an established method for low concentrations of pramipexole during release. With these results, it was shown that the drug release of pramipexole from the PVA matrix can be appropriately described by the LCW-UV. In the next step, a method was developed that can detect both the low pramipexole concentrations and the higher concentrated APIs during drug release that would all be incorporated in a polymedication in an ongoing study. Following the approach of Wollmer and Klein, who quantified levodopa and benserazide with two other API, a new chromatographic method was developed [43].

### 3.3. Simultaneous Quantification of Levodopa, Benserazide and Pramipexole in Dissolution Testing 

**Figure 7:**
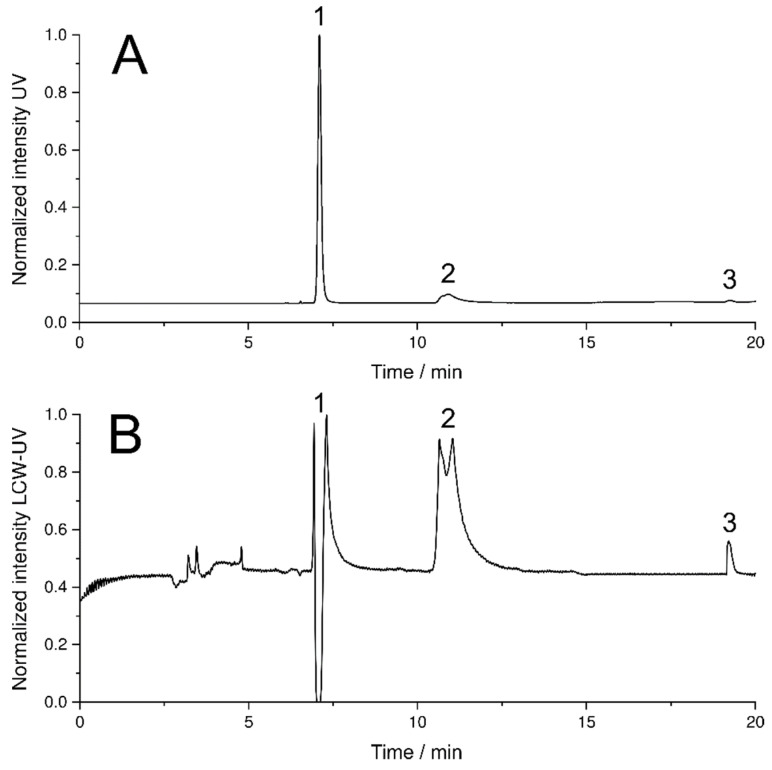
HPLC chromatogram using UV detection (**A**) and LCW-UV detection (**B**) of released levodopa (1), benserazide (2) and pramipexole (3) during dissolution study.

For the quantitative evaluation of the amounts of LD, BZ and P in dissolution studies, a new HPLC method was developed that enabled separation and quantification of all three APIs (method 2, described in Section 2.4). Levodopa eluted first at a retention time (*R_t_*) of 7.1 min with a peak width of 0.5 min, followed by benserazide (*R_t_*: 10.9 min) with a peak with of 1.5 min and pramipexole (*R_t_*: 19.3 min) with a peak width of 0.4 min (Figure 7A).

**Figure 8:**
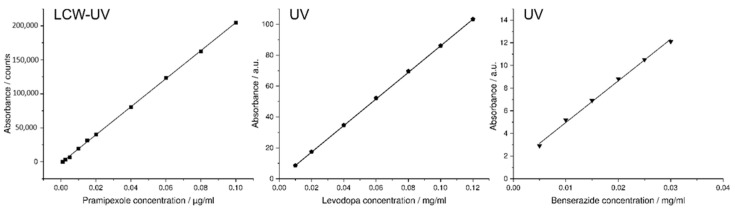
Calibration curve of pramipexole measured with the help of the LCW-UV detection system (1–100 ng/mL); calibration curves of benserazide (5–30 µg/mL), and levodopa (10–1200 µg/mL) both examined by UV detection.

The extended flow line of the LCW-UV did not result in an additional measurable dead time (Figure 7B), but the peak widths increased greatly due to the long light path, particularly for levodopa and benserazide, which emphasizes the importance of the separation of both substances from pramipexole by more than 5 min. In the chromatogram of the HPLC run obtained by UV detection, the peak of levodopa shows a narrow peak width. In the chromatogram resulting from LCW-UV measurements, the elution of levodopa is shown as a peak splitting signal. The peak shown negatively in the chromatogram indicates that for a short time more light is detected, but this is caused by saturation of the signal between 262 and 268 nm. Quantitative evaluation of levodopa is not possible using the LCW-UV method but is performed using the UV detector integrated with the HPLC. For the quantitative evaluation of benserazide, the UV detector is more suitable than the new implemented detector. The calculation of the LLOQ of pramipexole is involved the measurements of the UV and LCW-UV detector. By calculating the S/N, the LLOQ (S/N = 10) resulted in a concentration of 20 ng/mL (UV) and 1 ng/mL (LCW-UV). Compared to the first HPLC method, the LLOQ was subsequently lower for both LCW-UV and UV detection due to the focusing of pramipexole by the stepwise gradient of the second HPLC method. Again, an improvement of the quantification limit of the LCW-UV detection compared to UV detection after applying HPLC method 2 was achieved by a factor of 20. Calibration curves were also successfully established for LD (0.01–0.12 mg/mL) and BZ (0.005–0.03 mg/mL) by UV measurements. Figure 8 shows the resulting linear ranges of the three APIs.

**Figure 9:**
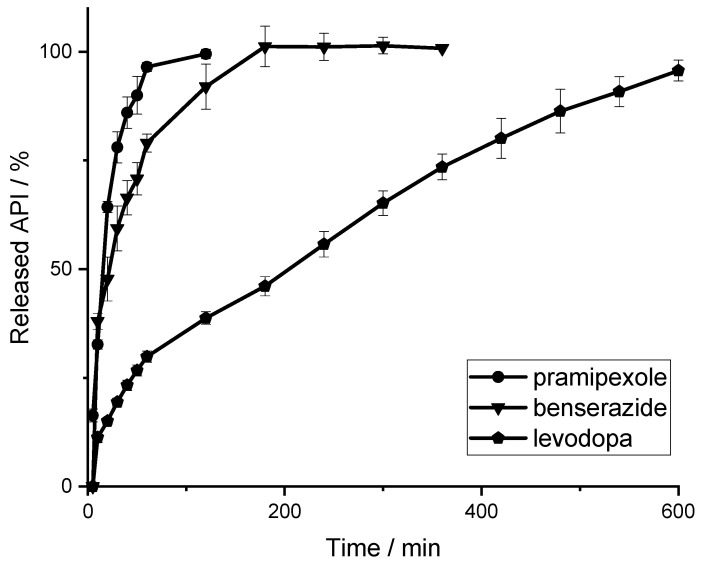
Release curve of 0.5% (*w/w*) pramipexole filaments ($\bar{m}$ = 17.51 mg) and levodopa/benserazide (40/10% *(w/w))* filaments ($\bar{m}$ = 250.7 mg) determined with LCW-UV (P) and UV (LD, BZ) measurement (*n* = 6, $\bar{x}\;\pm$ SD).

After the identification of the calibration curves, a second drug release study was performed as described in Section 2.2. Attempts were again made to represent the real dose for pharmacotherapy of the respect API, so the filament amount was taken to examine 88 µg of pramipexole, 25 mg of benserazide and 100 mg of levodopa. Since the filament length of the EVA-LD-BZ filament exceeded the size of the basket, the filament was cut into four shorter pieces. Thus, five filaments (one PVA-P filament stick, four EVA-LD-BZ filament sticks) were placed in the baskets. The dissolution profile of 0.5% (*w/w*) pramipexole filaments and filaments containing 40% (*w/w*) levodopa and 10% (*w/w*) benserazide are shown in Figure 9. Both the drug release of pramipexole and benserazide can be described according to first order kinetics. Within 80 min, 100% of the pramipexole was released from the PVA matrix and could be categorized as unmodified drug release. For benserazide, 100% of the drug was released from the insoluble EVA matrix after 180 min. For the third API, levodopa, the drug release from the EVA matrix can be described by Higuchi (square root) kinetics since the diffusion distance to be passed by the API through the matrix does not remain constant but increases steadily. The authors assume that levodopa and benserazide are not homogeneously distributed in the matrix consisting of VA and EVA, leading to the different drug release behaviors of LD and BZ. It is further speculated that the pore former VA might be the reason that the affinity of the distribution differs for the two API. This assumption would lead to the conclusion, that benserazide would have a higher affinity for VA than for EVA. In addition, the slightly better water solubility of BZ may also lead to the faster release. Further dissolution studies and formulation development will be performed as part of an ongoing study to obtain the required release kinetics. However, this study showed that all three APIs can be detected during drug release.

## 4. Conclusions

In this study, an LCW was applied as a flow cell to increase the light path using total internal reflection. For pramipexole, the LLOQ was improved by a factor of 20 with LCW-UV detection compared to conventional UV detection. With the help of this method, concentrations of 1–100 ng/mL, which occur during drug release of low-dosed pramipexole preparations, were detected simultaneously with two other released higher-dose APIs by UV detection. The calculated *f*_2_-value for comparison of LCW-UV vs. LC-MS/MS was >90, indicating that similar results for both technologies were obtained. The MDT was comparable for both methods (20 ± 0.4 min). The new method offers a promising alternative to expensive and time-consuming analytical technologies and can be easily integrated into existing HPLC systems. Especially during the development phase of individualized drug preparations and drug products of levodopa, benserazide and pramipexole, this analytical method can provide fast results after dissolution studies. The integration of the LCW-UV detection system can be used for various formulated API–polymer combinations and also drug products, e.g., 3D printed tablets, where the API dosing differs substantially regarding therapeutic regimens. 

## Figures and Tables

**Table 1:** Composition of filaments.

APIs and Excipients	Concentration in % (*w/w*)	Function
Formulation 1 (PVA-P):		
Pramipexole 2 HCl·H_2_O (P)	0.5	API
Polyvinyl alcohol (PVA)	99.5	matrix
Formulation 2 (EVA-LD-BZ):		
Levodopa (LD)	40	API
Benserazide HCl (BZ)	10	API
Poly (ethylene-vinyl acetate)-copolymer (82:18) (EVA)	35	matrix
Poly (vinylpyrrolidone-vinyl acetate)-copolymer (60:40) (VA)	15	pore former

**Table 2:** Adjusted temperatures and screw configuration of performed extrusions.

Temperature Profile in Zones 2–10/°C									
Zone	2	3	4	5	6	7	8	9	10
PVA-P formulation	30	100	180	180	180	180	180	195	195
EVA-LD-BZ formulation	30	180	190	200	220	220	220	220	220
Screw Configuration (Die–Gear)									
PVA-P/ EVA-LD-BZ formulation	die–10 CE 1 L/D–KZ 1: 5 × 60°–3 × 30°–5 CE 1 L/D–KZ 2: 4 × 90°–5 × 60°–3 × 30°–16 CE 1 L/D–2 CE 3/2 L/D–1 L/D adapter–gear								

CE = conveying element, KZ = kneading zone.

## Data Availability

The data presented in this study are available upon request from the corresponding author.

## Abbreviations

API	active pharmaceutical ingredient
BZ	benserazide
EVA	poly (ethylene-vinyl acetate)-copolymer
DAD	diode array detection
HME	hot melt extrusion
HPLC	high-performance liquid chromatography
LCW	liquid-core waveguide
LD	levodopa
LLOQ	lower limit of quantification
MDT	mean dissolution time
MS	mass spectrometry
P	pramipexole
PVA	polyvinyl alcohol
SD	standard deviation
S/N	signal-to-noise ratio
UV	ultraviolet
VA	poly (vinylpyrrolidone-vinyl acetate)-copolymer
